# Fractures’ associated mortality risk in orthogeriatric inpatients: a prospective 2-year survey

**DOI:** 10.1007/s41999-020-00392-1

**Published:** 2020-09-18

**Authors:** Andreas Wiedl, Stefan Förch, Annabel Fenwick, Edgar Mayr

**Affiliations:** Abteilung für Unfallchirurgie, Orthopädie, Plastische und Handchirurgie, Universitätsklinikum Augsburg, Stenglinstraße 2, 86156 Augsburg, Germany

**Keywords:** Orthogeriatrics, Mortality, Osteoporotic fragility fractures, Hip fractures, Vertebral fractures, Upper extremity fractures

## Abstract

**Purpose:**

The most common osteoporotic fragility fractures are hip, vertebral and upper extremity fractures. An association with increased mortality is widely described with their occurrence. Fracture-specific associated death rates were determined in a 2-year follow-up for patients treated on an orthogeriatric ward. These were compared amongst each other, examined for changes with age and their impact on the relative mortality risk in relation to the corresponding population.

**Methods:**

We assessed all patients that were treated in the course of a year on an orthogeriatric ward and suffered from the following injuries: hip (HF), vertebral (VF) and upper extremity fractures (UEF). In a 2-year follow-up it was possible to determine the month of death in the case of the patient’s decease. Pairwise comparisons of the three fracture type death rates were performed through Cox-Regression. We stratified the fracture-dependent absolute mortality and age-specific mortality risk (ASMR) for age groups 71–80, 81–90 and 91–95.

**Results:**

Overall, we assessed 240 patients with HF, 96 with VF and 127 with UEF over the span of a year. 1- and 2-year-mortality was: HF: 29.6% a.e. 42.9%, VF: 29.2% a.e. 36.5%, UEF: 20.5% a.e 34.6%. Pairwise comparisons of these mortality values revealed no significant differences. In association with HF and VF, we observed a significant increase of 2-year mortality for the oldest compared to the youngest patients (HF: 60.4% vs. 22.5%; *p* = 0.028) (VF 70% vs. 14.3%; *p* = 0.033). The analogue comparison for UEF revealed no relevant difference in age-dependent mortality (40.9% vs. 31.1%; *p* = 0.784). Common for all fracture types ASMR’s were more elevated in the younger patients and decreased with higher age.

**Conclusion:**

The fracture-related mortality in the 2-year follow-up was comparable. We observed a reduction of relative mortality risk in the oldest patients. While a direct influence of fracture on mortality must be supposed, we support the thesis of the fracture rather being an indicator of higher susceptibility of timely death.

## Key summary points

**Aim:**

To determine differences in mortality between hip-, vertebral- and upper extremity fractures of orthogeriatric inpatients and mortality’s respective age dependence.

**Findings:**

All fracture-related death rates were comparable and not significantly different. Age stratification showed a reduction in relative death risk at higher age in all groups.

**Message:**

The fracture event was an indicator of higher susceptibility of death in relatively younger orthogeriatric patients independent of fracture entity.

## Introduction

Osteoporotic fragility fractures are a common threat to older people. Worldwide, the total incidence of osteoporotic fractures is estimated around about 9 million every year [1]. Major types of fragility fractures include hip fractures, vertebral fractures and fractures of the upper limb (humeral and forearm fractures) [2]. Not only do these injuries cause pain, they also induce loss of function and mobility [3]. The risk for subsequent fractures is also raised by a single initial fracture. The mentioned injuries are well known to be accompanied by an increased mortality [4–9], which is assumed to be a sum of the baseline death rate of the corresponding age group due to comorbidity and age-related likeliness for decease and the excess mortality caused by the fracture itself [4, 10, 11]. The existing studies describe higher absolute mortalit*y* after hip and vertebral fractures in contrast to fractures of the upper limb [11, 12]. Common for all mentioned injuries, the relative risk of death in comparison to the corresponding age group shows a negative correlation with increasing age [4, 12]. The older the patient becomes, the less fracture-related mortality is observed. The influence of the fracture on the excess mortality was concluded to be higher for younger patients and was also found to be highest in the first year after the injury, decreasing with growing temporal distance to the initial fracture [4, 11, 12].

The aim of the actual study was to determine fracture-specific death rates of hip-, vertebral and upper extremity fractures in the context of an inward orthogeriatric treatment in a 2-year prospective follow-up. First, we wanted to point out differences in mortality amongst the mentioned fracture types. Second, we wanted to show the respective changes in absolute mortality depending on the patients’ age and display the relative mortality risk in comparison to the age-adjusted population.

## Materials and methods

We prospectively selected all patients that were treated in the course of a year from February 2014 to the end of January 2015, suffering from hip, vertebral, humeral and forearm fractures. There was a positive approval of the institutional review board of the Bavarian state chamber of medicine on the performance of this study (Sign: 7/11,192). Informed consent of patients and relatives was achieved*.* The inpatient treatment took place on a multidisciplinary orthogeriatric ward. The injury cause to admission was identified. Cofactors such as prehospital mobility and comorbidities were assessed via Parker-Mobility-Score (PMS) and Charlson-Comorbidity-Index (CCI). Follow-up was generated after 2 years sending questionnaires to patients and relatives. The primary subject of the examination was the month of death. Patients or relatives that did not answer our request via mail were contacted by telephone calls with a maximum of five attempts. Thereby we could determine the associated mortality*.* Patients with humeral and forearm fractures were united into the group “upper-extremity fractures” for simplification. Cox-regression showed a hazard-ratio of 1.0 (95% CI 0.49–1.86) comparing the mortality-risk of both groups. The outcome was assumed to be equivalent. We treated all hip fractures surgically, whereas vertebral fractures were integrated in a therapeutical algorithm that respected both morphology, clinical symptoms and the course of the treatment. This resulted in a fifty-fifty relation of conservatively to operatively treated fractures of the spine. Upper extremity fractures were preferably addressed through osteosynthesis in order to enable early functional treatment and preserve autonomy. Accordingly, the ratio between conservatively and surgically treated fractures was 1–5.

SPSS (IBM) was used for statistical analyses and calculations. Fisher’s exact test (FET) was performed to determine significance in the differences between 1- and 2-year-mortality. Kaplan–Meier curves were examined by the Log-Rank-Test (LRT). Finally, Cox-regression was used to describe the relative mortality risk between fracture groups and allowed to adjust for covariates such as gender, mobility via PMS and comorbidities via CCI. Each fracture group was, respectively, stratified into three subgroups by PMS and CCI: PMS 1–3, 4–6, 7–9 and CCI 0–1, 2–3, 4 and greater.

We elaborated both age-adjusted standardized mortality ratios (SMR) and age-specific mortality ratios (ASMR) for all fracture types. As reference, we used the mortality tables of the German Federal Statistical Office which were assessed using data from 2013 to 2015 [13], in order to calculate the corresponding 1- and 2-year-mortalities of the age-adjusted population*.* The ASMR was determined by distributing the patients into age groups. These were summarized for ascending age in 71–80 years (1), 81–90 years (2) and 91–95 years (3).

## Results

In the follow-up we assessed 240 (299 initially treated, 80.3% response rate) patients with primary hip, 96 (128 initially treated, 75.0% response rate) with vertebral and 127 (159 initially treated, 79.9% response rate) with upper extremity fractures. The hip fracture group’s mean age was 85.5 years and consisted of 58 (24.2%) men and 182 (75.8%) women. Vertebral fracture patients were on average 83.3 years old, distributed gender wise into 31 (32.3%) males and 65 (67.7%) females. 35 forearm fractures (mean age: 87.2; men: 5, 14.3%; women 30, 85.7%) and 92 humeral fractures (mean age: 84.2; men: 19, 20.7%; women: 73, 79.3%) were summed up to the group of upper extremity fractures (mean age: 85.1; men: 24, 18.9%; women: 103, 81.1%). The three mobility subgroups showed no significant difference in distribution amongst the fracture types (*p* = 0.635); neither did the three comorbidity subgroups (*p* = 0.253). We, therefore, assume a homogenous distribution regarding those parameters.

## Comparison of fracture-related mortality

**Fig. 1:**
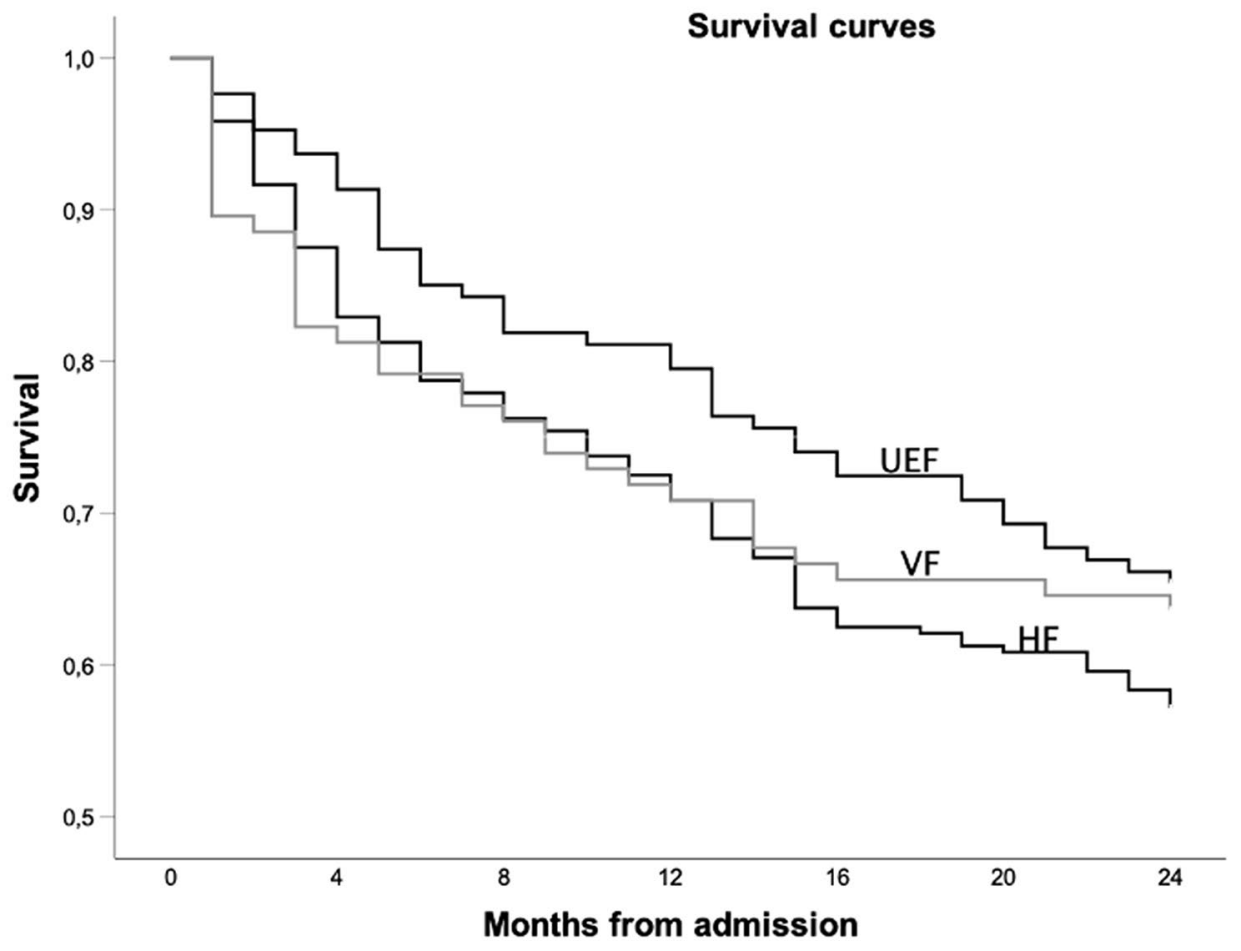
Survival curves of all fracture types

1- and 2-year-mortality was 29.6% and 42.9% after hip and accordingly 29.2% and 36.5% after vertebral fractures. Upper limb fractures were associated with the lowest mortality of 20.5% and 34.6% (Table 1). In the pairwise comparison, no statistic significances were identified concerning 1-year mortality (HF:VF *p* = 1.000 FET; HF:UEF *p* = 0.063 FET; VF:UEF *p* = 0.156 FET) or 2-year mortality (HF:VF *p* = 0.326 FET; HF:UEF *p* = 0.145 FET; VF:UEF *p* = 0.779 FET). Kaplan–Meier curves are illustrated in Fig. 1. The cofactors age, gender, mobility and comorbidity were included in the Cox-regression analysis to eliminate confounding variables. The risk ratio (hazard-ratio) of hip to vertebral fracture associated death was 0.95, a.e. of hip- to upper extremity fracture 1.27. The analogue relation between vertebral and upper limb fractures was 1.25. Table 2 shows 95% confidential intervals and *p* values. Although hip fractures showed the highest associated mortality followed by vertebral fractures and upper extremity fractures, there were no remarkable nor significant differences.

**Table 1.** 1- and 2- year mortality and statistical comparison of the fracture types

Mortality values	1-year-mortality	2-year-mortality
Hip fractures (HF)	29.6% (71/240)	42.9% (103/240)
Vertebral fractures (VF)	29.2% (28/96)	36.5% (35/96)
Upper extremity fractures (UEF)	20.5% (26/127)	34.6% (44/127)

Statistical testing	*p* values	*p* values
Fisher’s exact test		
HF: VF	1.000	0.326
HF: UEF	0.063	0.145
VF: UEF	0.156	0.779

**Table 2:** Hazard ratios in between the fracture-specific mortalities

	*p*	HR	95.0% CI for HR	
			Lower	Upper
Hip fractures: vertebral fractures	0.812	0.95	0.619	1.456
Hip fractures: upper extremity fractures	0.238	1.27	0.854	1.885
Vertebral fractures: upper extremity fractures	0.386	1.25	0.756	2.057

### Risk factors gender, mobility and comorbidities

**Fig. 2:**
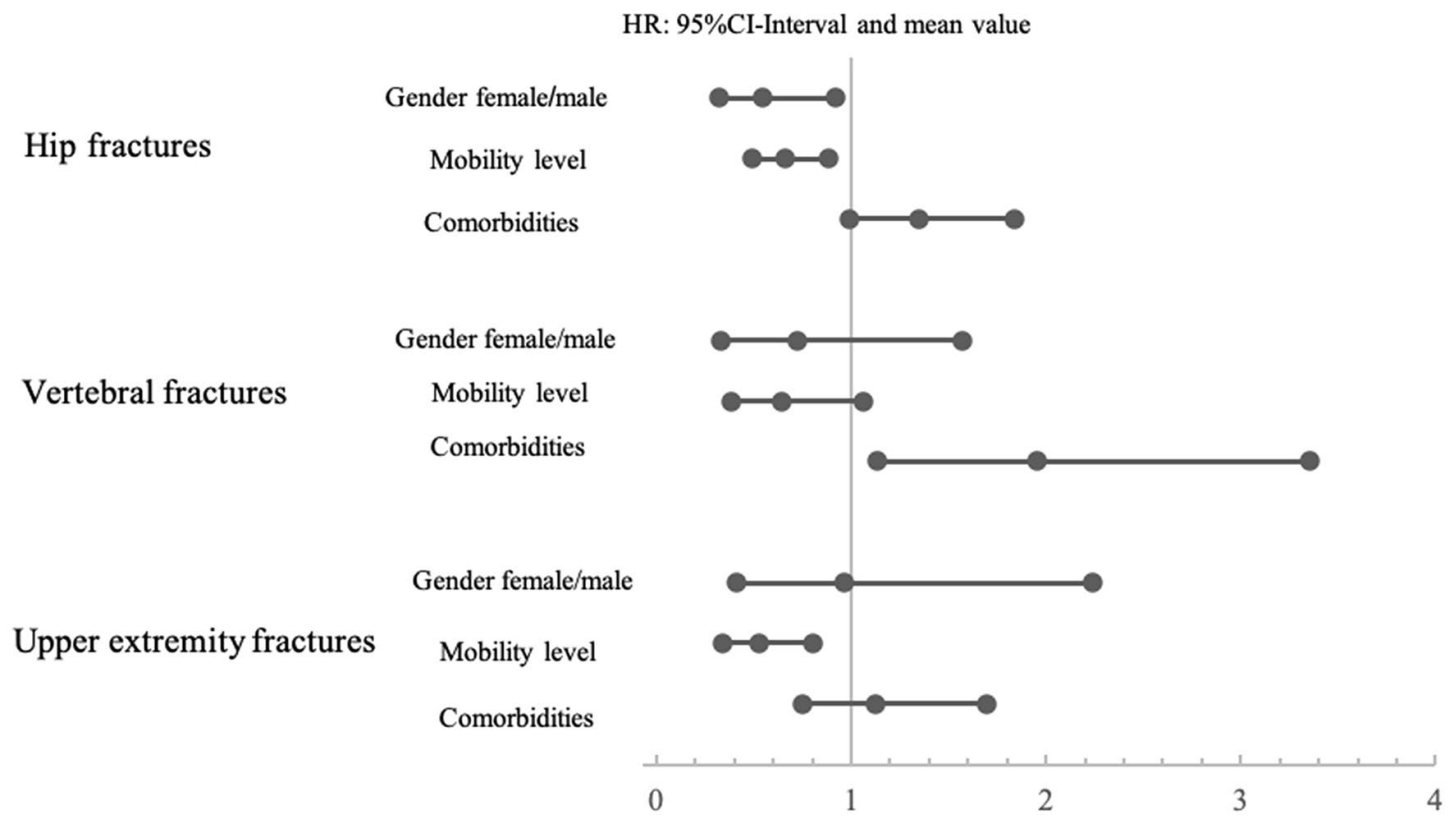
Hazard ratios and 95-CI intervals associated with risk factors for every injury: gender-related risk for female: male. Mobility-related risk for more mobile: less mobile. Comorbidity-related risk for more comorbidities: less comorbidities

We could identify gender and mobility as independent risk factors for mortality in the hip fracture group. Female patients displayed a HR of 0.55 (95% CI 0.324–0.921) compared to male patients. Significant differences in mortality between female and male patients were found neither after vertebral fractures calculating a HR of 0.72 (95% CI 0.330–1.570), nor after upper extremity fractures with a HR of 0.96 (95% CI 0.414–2.237). As the only independent risk factors we identified higher CCI in the vertebral and lower PMS in the upper extremity fracture groups. All HRs and corresponding 95% CI-intervals are displayed in Fig. 2.

### Age-dependent changes of mortality

Table 3 charts the mortality grouped by age and fracture type. Hip and vertebral fracture-associated 1- and 2-year-mortality showed a linear increase with higher age, being significantly different between group 1 and 3 (Table 3). In the upper extremity fracture cohort we observed no relevant increase of mortality in older patients.

**Table 3:** Age-dependent 1- and 2-year mortality for each fracture group

	Hip fractures		Vertebral fractures		Upper extremity fractures	
	1 year	2 years	1 year	2 years	1 year	2 years
Age groups						
71–80 (1)	12.5% (5/40)*	22.5% (9/40)**	14.3% (4/28)*	14.3% (4/28)**	17.9% (5/28)	31.1% (9/28)
81–90 (2)	28.1% (39/139)	42.4% (59/139)	30.9% (17/55)	40.0% (22/55)	21.9% (16/73)	35.6% (26/73)
91–95 (3)	43.8% (21/48)*	60.4% (29/48)**	60.0% (6/10)*	70.0% (7/10)**	22.7% (5/22)	40.9% (9/22)

Fracture-specific 1- and 2-year-mortalities for the age groups 71–80, 81–90 and 91–95. Significant differences are found between groups 1 and 3 in the hip- (*p=0.002, **p=0.028) and vertebral fracture (*p=0.01, **p=0.033) group

### Relative mortality risk in comparison to the population

All fracture entities were followed by a higher ASMR for age group 1 patients. Table 4 lists the ASMR’s for the stratified age groups and the age-adjusted SMR for each fracture type. The older the observed collective, the lower the ASMR. SMR and ASMR were lower after 2 years of follow-up than after one, which implicates a higher excess risk for death during the first year. The results for the SMR were comparable in between the injuries. Age group 1 was associated with the highest mortality ratios for each fracture type. Maybe due to the smaller sample sizes, only the hip fracture- and upper extremity fracture-associated ASMRs after 2 years showed significant 95%-CI intervals in this group. In age group 2 all mortality risks were significantly higher than that of the population, whereas in group 3 this effect persisted only for hip fractures. The mean ASMRs *in* group 3 of upper extremity fracture patients were 1.06, a.e. 1.05, which describes no relevant increased mortality risk.

**Table 4:** AMR and SMR for each fracture type including 95% confidential intervals

	Hip fractures		Vertebral fractures		Upper extremity fractures	
	1 year	2 years	1 year	2 years	1 year	2 years
Age-specific mortality ratios						
Age groups						
71–80 (1)	4.58 (0.57–8.59)	3.94 (1.36–5.52)	5.24 (0.10–10.38)	2.51 (0.05–4.97)	6.82 (0.84–12.80)	5.87 (2.04–9.70)
81–90 (2)	3.17 (2.18–4.16)	2.37 (1.76–2.98)	3.45 (1.81–5.09)	2.21 (1.29–3.13)	2.52 (1.28–3.76)	2.03 (1.25–2.81)
91–95 (3)	2.06 (1.18–2.94)	1.60 (1.02–2.18)	2.76 (0.55–4.97)	1.77 (0.46–3.08)	1.06 (0.13–1.99)	1.05 (0.36–1.74)
Age-adjusted standardized mortality ratios						
2.75 (2.08–3.42)		2.15 (1.72–2.58)	3.43 (2.14–4.72)	2.13 (1.39–2.85)	2.21 (1.36–3.06)	1.92 (1.35–2.49)

## Discussion

Approximately 460 patients were included in this study, the major part suffering from hip fractures. Compared to existing studies, we are talking about a moderate sample size here [4, 5, 11, 14]. We found no imbalance in the distribution of the functional status and present comorbidities amongst the injury types. The respective death rates were highest after hip, slightly lower after vertebral and lowest after upper-extremity fractures. Nevertheless, all analyses showed no significant differences comparing the injury-associated mortality values. Those in comparative terms high death rates associated to fractures of the upper extremity have not been observed before [4, 7, 11, 15, 16]. In contrast to our results, Johnell et al. reported a 1- and 2-year-mortality of 13%, a.e. 17% after shoulder and of 7% a.e. 11% after forearm fractures [12]. Nevertheless in the same study the according death rates for hip (22% a.e. 31%) and vertebral fractures (28% a.e. 40%) were comparable with our investigation [12]. Cauley et al. confirm these findings, describing similar mortality risks associated with hip and vertebral fractures and almost no increased mortality risk after forearm fractures [5]. In this context one study even found increased survival of patients with wrist fractures in comparison to their healthy peers [16]. Alarkawi et al. also described lower mortality of “non-hip non vertebral fractures” including upper extremity fractures in contrast to hip fractures [9]. Together with our observations, 1- and 2-year mortality after hip and vertebral fractures seems to be equivalent. In contrast to the mentioned publications, the reported mortality of 20.5% and 34.6% after humeral fractures and forearm fractures is not significantly different than after hip or vertebral fractures. A possible explanation of our finding is that the inpatient treatment of patients on the orthogeriatric unit could have selected an older, frailer and more multimorbid collective compared to the mentioned studies. Adam et al. calculated a similar 1-year mortality after the inward treatment of humeral fractures for patients aged 65 or older [17], which supports the previous presumption. It must also be considered that compared to our cohort, the existing literature describes a lower mean age of vertebral and especially upper extremity fractured patients [12, 18]. While our upper extremity fracture group displayed a mean age of 85, existing studies report respective cohorts being more than 10 years younger on average [9, 11, 12, 16]. At this age the absolute mortality risk might be less related to the mortality risk attributed to the fracture, which could explain why we observed no difference concerning mortality in between the fracture groups. Forearm and humeral injuries were merged in our analysis. As described in the methodical part of this article, the mortality in both groups was almost the same. Consequently, we suppose that there is no bias having summed up both entities in the analysis, as long as we did not miss out unknown confounding effects. An additional explanation to the high observed death rates would be that old patients often require walkers, crutches and generally their arms for mobilization. Therefore immobilization could result from upper extremity fractures and be causal for higher death rates. This consideration is supported by the relevant influence of preexisting mobility in our analyses. We observed a significantly elevated mortality risk of men in the hip fracture group, no significant but slight increase in the vertebral fracture group and no difference in the upper extremity fracture group. Increased male mortality after hip fractures has been widely observed and published [19–22]. Center et al. and Johnell et al. also described higher death rates in men after all osteoporotic fractures including upper extremity fractures and vertebral fractures, partly without significance [4, 12].

The absolute mortality rose with every age group after vertebral and hip fractures almost proportionally. The literature can confirm this trend [4, 12, 21, 23] and it seems easy to understand that survival rate decreases with age. That is why it is more interesting that upper extremity fracture-associated death rates seemed to stay quite stable among the age groups. In contrast, Johnell et al. showed remarkably increased mortality after shoulder and forearm fractures in older men and women, comparing age 60–80 [12]. Accordingly, Shortt et al. identified age as an independent risk factor in both proximal humeral and wrist fractures [16]. The observation of mortality being almost age-independent after injuries of the upper extremity corresponds to our observation of a sharp reduction of the relative mortality risk with higher age as it will be discussed below.

The age-specific mortality ratio (ASMR) can be seen as the additional risk of death due to the fracture event. It was highest for the youngest patients between 71and 80 years for all injuries and among those in upper extremity fracture patients. As the age-stratified mortality in the latter group remained stable, the respective ASMR dropped to approximately 1 for patients in between 91 and 95 years and, therefore, no excess risk of death was observed. Hip and vertebral fracture-associated ASMRs sank with advancing age but did not fall to one and thus mortality risk did not reach the population’s baseline. Common for all examined osteoporotic injuries, the 2 years’ follow-up ASMRs and the SMRs were lower than after 1 year. The relative risk of death was sinking with temporal distance to the trauma, being highest in the first year, which has been also approved in literature [4, 11, 12]. Studies consistently report age-related drops in the relative mortality risk of both hip and vertebral fractures with residual increased risk for the oldest patients [12, 18, 23]. This has also been shown for humeral fractures and wrist fractures. Specifically, humeral fracture-associated mortality risk was described to drop to the population’s baseline in the oldest collective [12]. Accordingly, observations showed no difference to the population’s death risk after forearm fractures in all age groups [4, 12]. All investigations point out that age- and comorbidity-related mortality has more importance for the oldest patients than fracture-related mortality. Consequently, this suggests a higher excess mortality in younger patients. We would like to discuss two main explanations of increased mortality resulting from fragility fractures. On one hand, it is attributable to the injury and its consequences like immobility, loss of function, complications and surgical interventions [24]. On the other hand, the fracture itself is just a byproduct of general frailty, morbidity and predisposition of timely death. Accordingly, fragility fractures might be symptomatic for patients that are more likely to decease earlier. In our observation, the remarkable drop in mortality risk for the oldest indicate a not negligible fracture independent influence on mortality. Large examinations, including high patient counts, control samples and their functional, comorbid and general health levels, must be assessed to really measure the weight of the fracture in this equation and find the main culprit of fracture-related increased mortality.

We did not deliver the therapeutic influence on mortality. As described in the methodical part, vertebral and upper extremity fracture groups consisted both of conservatively and surgically treated patients. Surgically treated thoracolumbar vertebral fractures were distinctly but not significantly associated with increased survival, whereas no significant differences were observed between upper extremity therapy groups (in publication). A detailed disclosure would go beyond the scope of this article.

This study has some limitations. The total patient count was moderate compared to existing studies. Especially the oldest age group of ages 91–95 years was small. We examined only inward patients that were treated on an orthogeriatric unit. The admission was assessed by specific criteria as age, mobility, cognitive status, vision or acoustical impairments and polypharmacy. Thereby not every patient suffering from the examined fragility fractures was included, which can have caused a selection bias. The higher mortality of upper extremity fractures could have been influenced by the fact of having examined a collective with generally increased risk which is more independent from fracture-associated mortality risk. This possibility is underlined by the fact that the according inpatient-treated upper extremity fracture cohort was remarkably older than those in other studies as already mentioned above. Furthermore, we did not acquire an age-adjusted control group and could thereby not differentiate the weighted influence of fracture and other variates.

After the recruitment ended 2017, large statistical analyses lead to a delay in report. By affecting ASMR and SMR fluctuations of the general population's survival rates over the last years implicate distracted results, if e.g patient cohorts from 2014 were compared to the population's data from 2019. In order to safely eliminate this bias, we acquired the epidemiological data and death rates from the German Federal Statistical Office’s published “periodic death tables” of the years 2013–2015 [13]. The mentioned periodic death tables were the most appropriate to our assessment period from February 2014 to January 2015.

The count of our patient group was strong enough to distinguish age-dependent differences and identify significant variables. Additionally, we included the main influential confounders on mortality. Our analyses furthermore qualify very good for comparisons to other studies concerning orthogeriatric inpatient collectives.

We observed no significant differences in mortality amongst the major fragility fractures of the hip, the spine and the upper limb. Although being comparable, hip fracture mortality was highest followed by vertebral and upper extremity fractures. While hip and vertebral fracture-related death rate increased with the patient’s age, it did not for upper extremity fractures. Compared to the general population the highest mortality risk was expounded in the youngest patients for all fracture types and mostly for upper extremity fractures. The relative mortality risk sank with higher age and longer observation period. The excess risk is more pronounced in younger patients; maybe in this context fractures occur in multimorbid pre-aged patients. Consequently, we assume a major importance of fragility fractures in being an indicator for predisposition of timely death due to comorbidities and frailty.
